# Histone acetylation regulates the expression of genes involved in worker reproduction in the ant *Temnothorax rugatulus*

**DOI:** 10.1186/s12864-021-08196-8

**Published:** 2021-12-03

**Authors:** Marina Choppin, Barbara Feldmeyer, Susanne Foitzik

**Affiliations:** 1 Institute of Organismic and Molecular Evolution, Johannes Gutenberg University, Mainz, Germany; 2 Senckenberg Biodiversity and Climate Research Centre (SBiK-F), Molecular Ecology, Senckenberg, Frankfurt, Germany

**Keywords:** Gene regulation, epigenetics, phenotypic plasticity, reproduction, ants

## Abstract

**Background:**

In insect societies, queens monopolize reproduction while workers perform tasks such as brood care or foraging. Queen loss leads to ovary development and lifespan extension in workers of many ant species. However, the underlying molecular mechanisms of this phenotypic plasticity remain unclear. Recent studies highlight the importance of epigenetics in regulating plastic traits in social insects. Thus, we investigated the role of histone acetylation in regulating worker reproduction in the ant *Temnothorax rugatulus*. We removed queens from their colonies to induce worker fecundity, and either fed workers with chemical inhibitors of histone acetylation (C646), deacetylation (TSA), or the solvent (DMSO) as control. We monitored worker number for six weeks after which we assessed ovary development and sequenced fat body mRNA.

**Results:**

Workers survived better in queenless colonies. They also developed their ovaries after queen removal in control colonies as expected, but not in colonies treated with the chemical inhibitors. Both inhibitors affected gene expression, although the inhibition of histone acetylation using C646 altered the expression of more genes with immunity, fecundity, and longevity functionalities. Interestingly, these C646-treated workers shared many upregulated genes with infertile workers from queenright colonies. We also identified one gene with antioxidant properties commonly downregulated in infertile workers from queenright colonies and both C646 and TSA-treated workers from queenless colonies.

**Conclusion:**

Our results suggest that histone acetylation is involved in the molecular regulation of worker reproduction, and thus point to an important role of histone modifications in modulating phenotypic plasticity of life history traits in social insects.

**Supplementary Information:**

The online version contains supplementary material available at 10.1186/s12864-021-08196-8.

## Background

Eusocial insect societies exhibit a reproductive division of labor where one or a few females (often called queens) reproduce, whereas workers perform all other tasks including brood care, nest defense, and foraging [1]. Workers thus sacrifice their own reproduction and this evolutionary incongruity is commonly explained by Hamilton’s inclusive fitness theory, which states that genes of sterile altruists can be transmitted indirectly to the next generation by helping closely related reproductives [2]. The proximate mechanisms underlying the maintenance of worker sterility in social insect colonies have been extensively investigated. Worker reproduction is regulated via chemical signals emitted by the queen or her brood [3–5], or through social control meditated by the queen or by the workers themselves [6–8]. However, following the loss of their queen or even sometimes in queenright colonies, social insect workers can circumvent those restraints and successfully gain direct fitness benefits by laying haploid, male-destined eggs [9–13]. Reproduction has strong effects on the physiology and immunity of workers, who can become more resistant to oxidative stress and often live longer [14–17]. These positive effects of reproduction have been linked to the activation of signaling pathways such as the insulin/insulin-like growth factor (IIS), the target of rapamycin (mTOR), and the alpha-ketoglutarate (alpha-KG) [18]. In fact, gene expression changes profoundly in workers after queen loss in social wasps [19], honeybees [20], and ants [18, 21] and in various tissues from the brain to the fat body. Similarly, gene expression differs between reproductive and sterile bumblebee workers [22]. Worker fecundity thus appears to be a highly plastic trait positively linked to lifespan [23]. This opens up exciting new avenues to study the molecular regulation of plasticity in fecundity and longevity in social insect workers [24]. Indeed, the transcriptomic changes linked to worker reproduction have been well characterized, while the underlying gene regulatory mechanisms remain largely unexplored.

Epigenetic mechanisms including DNA methylation and histone modifications have been proposed to play a major role in the extraordinary phenotypic plasticity exhibited by social insects [25–31]. In Carpenter ants, histone modifications have been associated with behavioral differences between major and minor ant workers [32–34] and worker polymorphism [35]. Histone acetylation has also been associated with the ability of workers to adjust to new daily rhythms [36]. Besides, there is growing evidence for the role of histone modifications in caste differentiation. In honey bees, queen development is largely controlled by royal jelly, a secretion that has histone deacetylase inhibitor (HDACi) activity [37]. Moreover, caste-determined female larvae exhibit genome-wide differences in histone acetylation and methylation patterns, which are linked to caste-specific gene expression [38]. Besides, the transition of non-reproductive to reproductive workers (also called gamergates) has been associated with transcriptomic changes linked to epigenetic pathways in the ant *Harpegnathos saltator* [39].

In this study, we used the ant *Temnothorax rugatulus* to investigate the role of histone acetylation in the regulation of worker reproduction following the loss of their queen. This common Myrmicine ant builds small nests of 50 to 2000 workers with one to several queens and evolved two queen morphs, the large macrogynes, and the small microgynes, associated with alternative reproductive strategies [40–42]. Queens can live over ten years and their gene expression in the brain and fat body changes with age [43]. Following queen loss, *T. rugatulus* workers are known to develop their ovaries, start laying haploid eggs, live longer, and show transcriptomic changes in the fat body, a physiologically active tissue [14, 18]. Here, we asked whether histone acetylation is required for workers to plastically respond to queen loss by altering their ovary development and associated gene expression. We used queen removal to induce fecundity in workers while feeding them with chemical inhibitors of histone acetylation (C646) or deacetylation (Trichostatin A; TSA). Based on previous studies, we predicted that workers would develop ovaries, survive better, and show transcriptomic changes in the fat body following queen removal. If histone acetylation does play a role in the regulation of worker reproduction, we expected the chemical inhibitors C646 and TSA to prevent workers from developing their ovaries following queen removal, and to alter the expression of fecundity and longevity genes, preventing workers to reproduce and live longer following the loss of their queen.

## Results

### Effects of queen removal

#### Worker number and fecundity

**Fig. 1:**
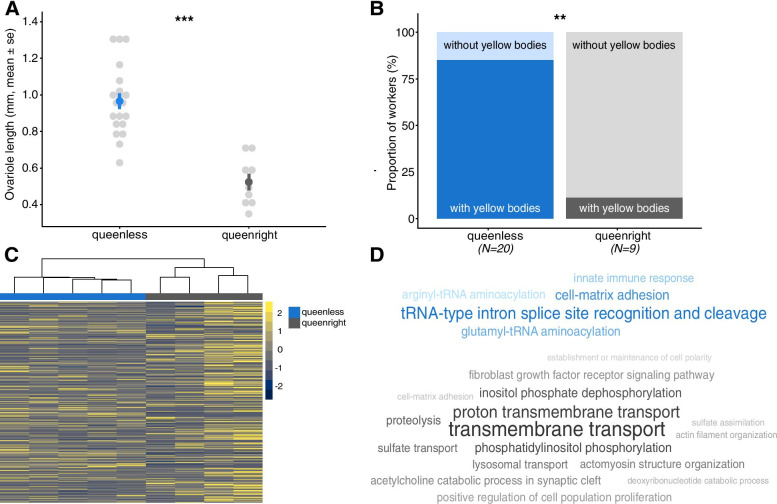
**A)** Effect of queen removal on ovariole length and **B)** the proportion of workers with yellow bodies in their ovaries at the end of the experiment at week 6. Levels of significance are indicated as follows: ** *p* < 0.01 and *** *p* < 0.001. **C)** Heatmap showing the expression levels of the differentially expressed genes between the “queenless” (control, blue) and “queenright” (grey) groups and the clustering of samples per group. **D)** Word clouds showing the overrepresented functions associated with downregulated (top, blue) and upregulated (bottom, grey) genes in the group “queenright”. In the word clouds, *p*-value significance is positively correlated with the size and shade darkness of the word

Worker number decreased less strongly in queenless compared to queenright colonies (interaction time x queen removal: X^2^ = 9.723, df = 2, *p* = 0.008; Additional file 1 Fig. S1). Workers from queenless colonies had longer ovarioles (X^2^ = 30.578, df = 1, *p* < 0.001; Fig. 1A), were more likely to have yellow bodies (X^2^ = 9.588, df = 1, *p* = 0.002; Fig. 1B), and also tentatively more likely to have white eggs in their ovaries (X^2^ = 2.828, df = 1, *p* = 0.093; Additional file 1 Fig. S2). Yellow bodies are small endocrine structures that remain in the ovaries after an egg has been laid, and thus provide evidence for reproductive activity [44–46].

#### Gene expression and functional enrichment

**Fig. 2:**
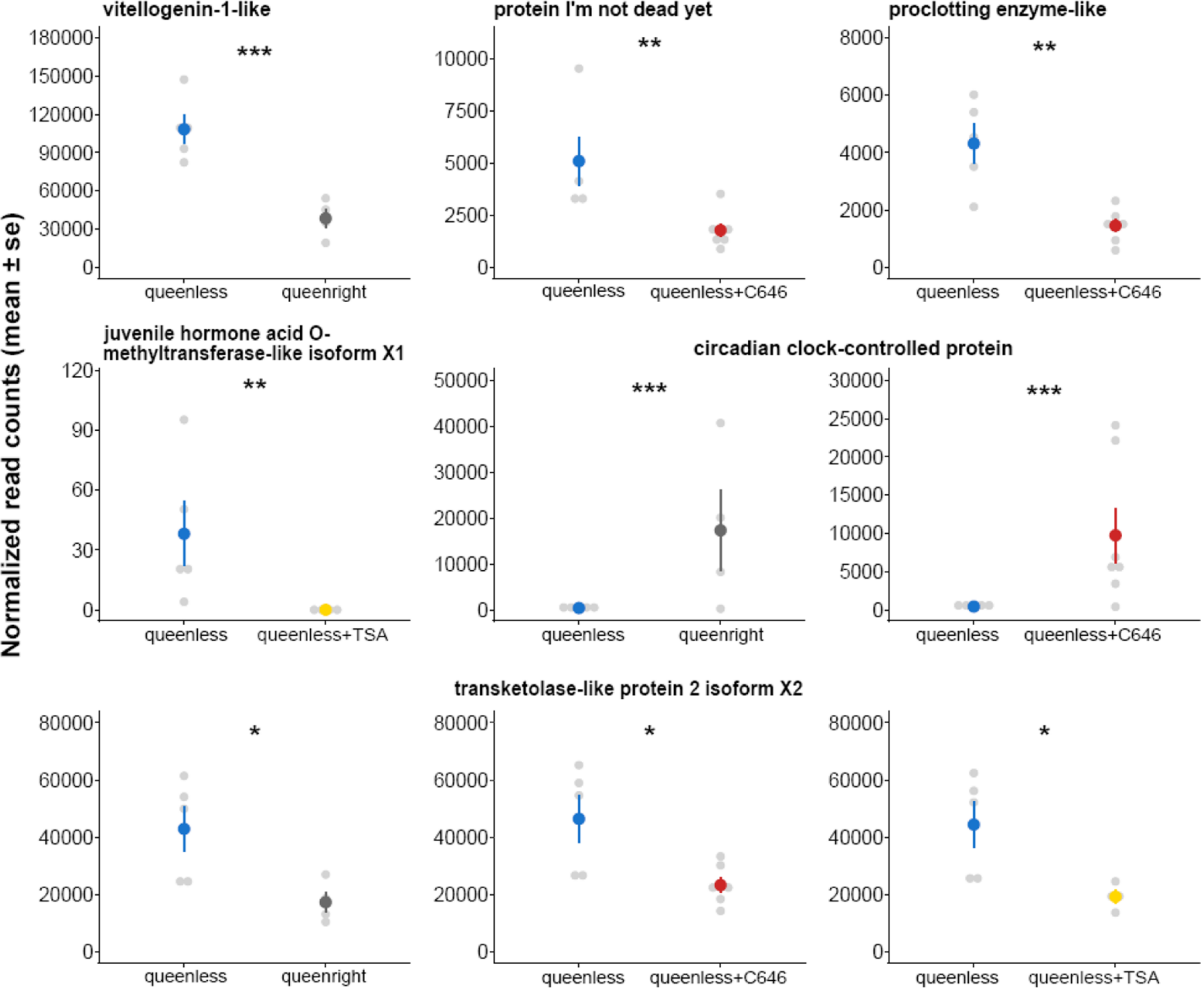
**First row:** expression levels of the genes “vitellogenin-1-like” (*p* < 0.001) downregulated in the group “queenright” compared to the group “queenless” (control), “protein I’m not dead yet” (*p* = 0.004) and “proclotting enzyme-like” (*p* = 0.001) downregulated in the group “queenless+C646” compared to the group “queenless” (control). **Second row:** expression levels of the genes “juvenile hormone acid-O-methyltransferase-like isoform X1” (*p* = 0.01) downregulated in the group “queenless+TSA” compared to the group “queenless” (control) and the gene “circadian clock-controlled protein” (p_QR_ < 0.001 and p_C646_ < 0.001) commonly downregulated in the groups “queenright” and “queenless+C646” compared to “queenless” (control). **Third row:** expression levels of the gene “transketolase-like protein 2 isoform X2” (p_QR_ = 0.036, p_C646_ = 0.031 and p_TSA_ = 0.045) commonly downregulated in the groups “queenright”, “queenless+C646” and “queenless+TSA” compared to “queenless” (control). Levels of significance are indicated as follows: * *p* < 0.05, ** *p* < 0.01 and *** *p* < 0.001

We found 346 differentially expressed genes (DEGs) between workers from the “queenless” and “queenright” groups (Additional file 2), among which 206 were upregulated and 140 downregulated in the queenright group. The samples clearly clustered according to treatment in the heatmap based on all DEGs (Fig. 1C). The enrichment analysis revealed that workers in presence of their queen downregulated genes related to five functions including “innate immune response” (Fig. 1D and Additional file 3). This is reflected by multiple immune genes in our list of top 15 downregulated genes in the queenright group including “FK506-binding protein 2 isoform X1” or “chymotrypsin-2-like”. Also, a gene coding for “vitellogenin-1-like” (Fig. 2) was found to be downregulated in workers from queenright colonies. As vitellogenin copies are so far annotated separately for each genome without orthology inference, the comparison of vitellogenins across species is difficult. Therefore, we investigated where our “vitellogenin-1-like” copy falls within the vitellogenin phylogeny by constructing a maximum likelihood phylogeny with RAxML [47] using sequences previously used in [48]. We found that it clusters close to the conventional vitellogenins (Additional file 1 Fig. S3), and thus refer to this vitellogenin copy as “conventional vitellogenin” in the rest of the manuscript. Queen presence also affected the expression of many regulatory genes, such as transcription factors including “zinc finger protein 454-like”.

### Effects of chemical inhibitors

#### Worker number, egg production, and fecundity

**Fig. 3:**
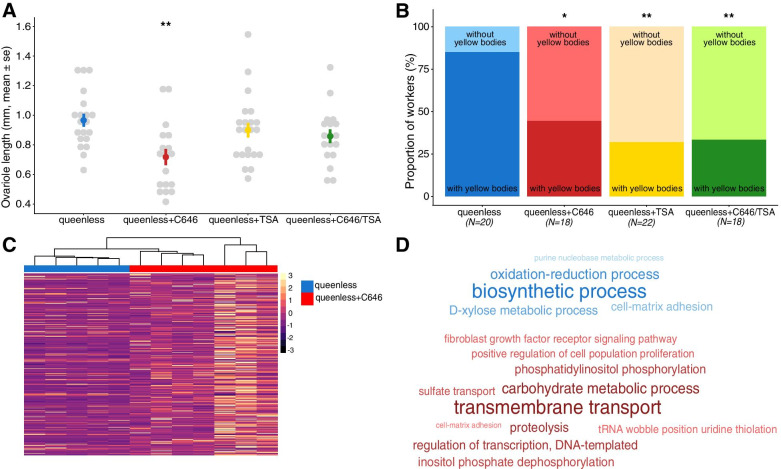
**A)** Effect of treatment on ovariole length and **B)** the proportion of workers with yellow bodies in their ovaries at the end of the experiment at week 6. Levels of significance are indicated as follows: * *p* < 0.05 and ** *p* < 0.01. **C)** Heatmap showing the expression levels of the differentially expressed genes between the “queenless” (control, blue) and “queenless+C646” (red) groups and the clustering of samples per group. **D)** Word clouds showing the overrepresented functions associated with downregulated (top, blue) and upregulated (bottom, red) genes in the group “queenless+C646”. In the word clouds, *p*-value significance is positively correlated with the size and shade darkness of the word

Here we focus on queenless colonies that were either fed with Dimethyl Sulfoxide (DMSO) only as control or additionally treated with C646, TSA, or both inhibitors. Again, worker number generally decreased over time (X^2^ = 719.732, df = 2, *p* < 0.001), but irrespective of treatment (X^2^ = 7.493, df = 6, *p* = 0.278; Additional file 1 Fig. S4). After six weeks, 23% of colonies had eggs and the presence of eggs was unaffected by treatment (X^2^ = 2.521, df = 3, *p* = 0.472; Additional file 1 Fig. S5). However, workers treated with the epigenetic inhibitors had shorter ovarioles (X^2^ = 11.569, df = 3, *p* = 0.009; Fig. 3A) and a smaller proportion of treated workers had yellow bodies in their ovaries (X^2^ = 9.721, df = 3, *p* = 0.021; Fig. 3B) compared to control workers. More precisely, C646-treated workers exhibited shorter ovarioles (lmer:t_C646_ = −3.290, p_C646_ = 0.002) and were less likely to have yellow bodies in the ovaries (glmer:z_C646_ = −2.366, p_C646_ = 0.018) compared to workers fed with DMSO only. In the TSA treatment, fewer workers had yellow bodies in their ovaries compared to control workers (glmer:z_TSA_ = −2.949, p_TSA_ = 0.003), although ovariole length was unaffected (lmer:t_TSA_ = −0.891, p_TSA_ = 0.379). None of the inhibitors affected the proportion of workers with white eggs in the ovaries (X^2^ = 5.817, df = 3, *p* = 0.121; Additional file 1 Fig. S6).

#### Gene expression and functional enrichment

We found 306 differentially expressed genes between workers from the control group and workers treated with the inhibitor of histone acetylation C646 (Additional file 2), among which 247 were upregulated and 59 were downregulated in the C646-treated workers. The heatmap revealed that C646 samples clustered well together (Fig. 3C). C646-treated workers downregulated genes with longevity functionalities such as “protein I’m not dead yet” also called “Indy” [49] (Fig. 2), or genes with an immune function like “proclotting enzyme-like” [50] (Fig. 2). Workers fed with C646 also downregulated seven genes related to the synthesis of fatty acids, versus only one in the control. As expected and corroborating the efficacy of our treatment, we found four histone-related genes upregulated in C646-treated workers (“histone H2A-like”, “histone H3”, “histone PARylation factor 1 isoform X2” and “late histone H1-like”). Our enrichment analysis revealed the overrepresentation of five functions associated with downregulated genes in C646-treated workers including “oxidation-reduction process” (Fig. 3D and Additional file 3).

Between workers from the control group and workers treated with the inhibitor of histone deacetylation TSA, we found 33 differentially expressed genes (Additional file 2). Three genes were upregulated and 30 were downregulated in the TSA-treated workers. The heatmaps created using the 33 DEGs revealed a good clustering of our samples by group (Additional file 1 Fig. S7). Based on the low number of DEGs between the two groups we only found the functions “transposition, DNA-mediated” and “autophagy” significantly overrepresented in the DEGs of the TSA-treated workers (Additional file 3). Although the TSA treatment had weaker effects on worker fecundity, we did find the aging and fecundity-associated gene “juvenile hormone acid O-methyltransferase-like isoform X1” [51, 52] downregulated in TSA-treated workers (Fig. 2).

### Overlapping genes between groups

**Fig. 4:**
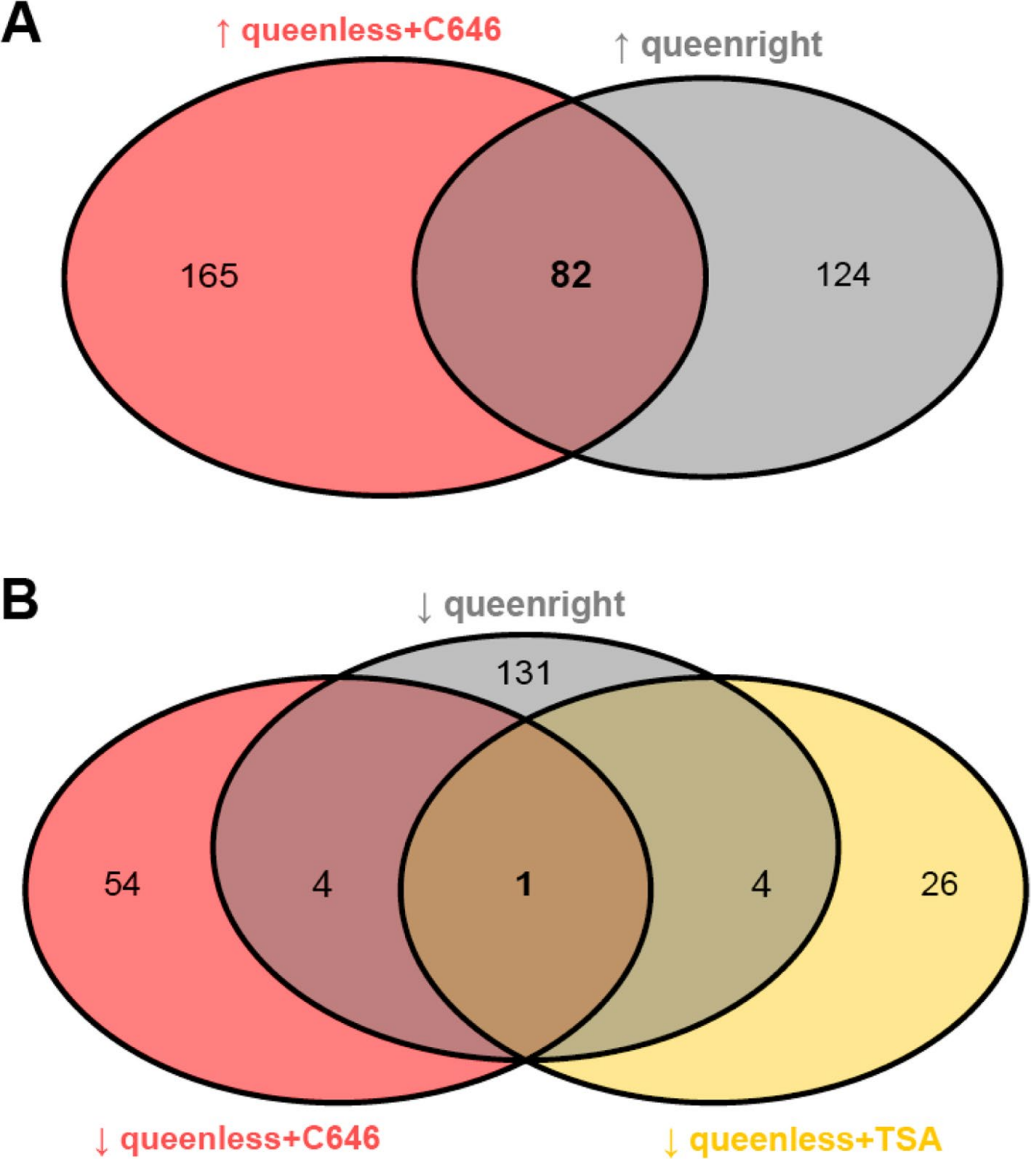
**A)** Venn diagram showing the number of genes singly upregulated in the groups “queenless+C646” (165) and “queenright” (124) and the number of genes commonly upregulated between these two groups (82). **B)** Venn diagram showing the number of genes singly downregulated in the groups “queenless+C646” (54), “queenright” (131), and “queenless+TSA” (26), the number of genes commonly downregulated between “queenright” and “queenless+C646” (4) or “queenless+TSA” (4) and the number of genes commonly downregulated between all three groups (1)

As indicated above, the chemical inhibitors of histone acetylation and deacetylation impaired worker ovary development following queen removal. Thus, we asked whether the transcriptomes of inhibitor-treated workers were similar to the ones of infertile workers from queenright colonies. Indeed, between the groups “queenright” and “queenless+C646” we found 82 genes commonly upregulated (Fig. 4A and Additional file 2) and five genes commonly downregulated (Fig. 4B and Additional file 2) in workers. We additionally found five genes commonly downregulated between the groups “queenright” and “queenless+TSA” (Fig. 4B and Additional file 2). These three numbers of overlapping genes were higher than expected by chance as evidenced by resampling random gene lists (Additional file 1 Fig. S8). Among the commonly upregulated genes between workers with a queen and C646-treated workers, we found genes associated with circadian rhythm like “circadian clock-controlled protein” (Fig. 2). We additionally found many genes associated with digestion like “mucin-5 AC-like”, “probable salivary secreted peptide” or “silk gland factor 1”. Finally, the gene “transketolase-like protein 2 isoform X2” coding for an enzyme with antioxidant properties [53–55] was commonly downregulated in all three groups in comparison to the control (Fig. 2 and Fig. 4B).

## Discussion

In this study, we investigated the role of histone acetylation in the regulation of genes associated with worker reproduction in the ant *Temnothorax rugatulus*. We removed queens from their colonies while feeding the workers with chemical inhibitors of histone acetylation (C646) or deacetylation (TSA). Our phenotypic and transcriptomic results confirmed that worker fecundity and survival increased after queen removal, as shown before in this species [14, 18]. However, the ovary development of workers from queenless colonies was impaired when treated with the chemical inhibitors, although their survival was unaffected. On a molecular level, the inhibition of histone acetylation using C646 had a stronger effect than the inhibition of histone deacetylation using TSA, as shown by the difference in the number of differentially expressed genes (306 vs 33). Yet, workers from both treatments downregulated many genes related to fecundity, immunity, and longevity, compared to the queenless control. More importantly, a large number of genes were commonly upregulated between infertile workers from queenright colonies and C646-treated workers from queenless colonies, pointing to similarities in gene expression. We additionally found one gene with antioxidant properties commonly downregulated between infertile workers and both C646 and TSA-treated workers, in comparison to workers in the DMSO control, which might be linked to the extended survival of these fecund workers.

Ant workers start to reproduce and live longer after the loss of their queen in many ant species [11, 15, 56] including our model *Temnothorax rugatulus* [14, 18]*.* We took advantage of this plasticity by inducing fecundity in workers using queen removal and confirmed that workers from queenless colonies developed their ovaries, started laying eggs, survived better, and shifted their gene expression in the fat body. Our transcriptomic analysis revealed the upregulation of a conventional vitellogenin (vg) in fecund workers from queenless colonies. During social insect evolution, vg genes underwent several duplications followed by diversification and sub-functionalization. Various vg orthologues now take over different functions in behavior [48] and physiology, including fecundity [57] and aging [58], in social insects. Nevertheless, conventional vitellogenins, like the vitellogenin copy we found, have an ancestral fecundity function as an egg yolk precursor [57] rather than a derived role, as in worker caste differentiation [59, 60]. Additionally, the expression of conventional vitellogenins has been linked to oxidative stress resistance in honeybee workers [61]. Besides, another study on *T. rugatulus* detected an upregulated vg receptor in fecund workers [14], pointing to the importance of vg-associated pathways for worker reproduction.

Longevity is traded off with immunity in many organisms due to the cost of an active immune system and the production of reactive oxygen species during immune reactions [62, 63]. Nevertheless, our results show that the longer-lived, fecund workers from queenless colonies activate genes with an innate immune response functionality, which is consistent with previous findings in *Temnothorax rugatulus* showing that fecund workers express more immunity genes following an immune challenge, compared to infertile workers [14]. Similarly, the highly fecund young queens of *T. rugatulus* upregulate immune genes in the Toll-pathway [43], which plays a crucial role in insect immunity [64]. These findings provide evidence that fecund ants invest in a longer lifespan and immunity concurrently, supporting other studies on social insects indicating that life history trade-offs have shifted during their social evolution [65, 66].

Interestingly, ant workers treated with chemical inhibitors following queen removal had a lower ovary development and were less likely to exhibit yellow bodies, which provide evidence for egg-laying [44–46], compared to control workers from queenless colonies. This indicates that dynamic changes in histone acetylation might be required for workers to shift to fecund phenotypes. In social insects, histone acetylation has previously been linked to various processes including the regulation of foraging behavior and caste determination [33, 34, 37, 38], but evidence for the regulation of life history traits such as fecundity and longevity have been lacking so far. Meanwhile, in solitary insects such as the pea aphid, the inhibition of histone acetylation and deacetylation affects development, fertility, and longevity [67]. In fact, fertility appears to be regulated by this epigenetic mark in various taxonomic groups, which includes other insects such as the planthopper *Nilaparvata lugens* [68], but also mammals including mice [69] and men [70].

The inhibition of histone acetylation using C646 had more severe consequences on gene expression in worker fat bodies compared to the inhibition of histone deacetylation using TSA, shifting the expression of nearly 10 times as many genes. Among the most strongly downregulated genes in workers treated with the chemical inhibitors, we found interesting candidates such as the Indy protein, which has been linked to longevity in *Drosophila* [49]. In these solitary insects, *Indy* knock-outs show extended lifespans, while we found this gene to be upregulated in fecund, longer-lived workers. This reversed link to fecundity and longevity might be due to shifts in gene networks underlying these life history traits in social insects [43, 71]. We additionally found the enzyme proclotting, involved in the innate immune response [50], and a gene associated with juvenile hormone, which plays a role in many physiological processes including aging [51]. We also detected seven downregulated genes associated with the fatty-acid synthesis in the queenless, C646-treated group compared to only one in the queenless control. Fatty acids are involved in the synthesis of cuticular hydrocarbons [72], and reproductive and non-reproductive individuals exhibit different odors in social insects [73, 74] including *Temnothorax* ants [75]. Besides, many histone-related genes were upregulated in the group where histone acetylation was inhibited (4/247) compared to our control (0/59), attesting to the efficacy of our treatment on a molecular level. Chromatin Immunoprecipitation sequencing (ChIP-sequencing) will be our next logical step to both confirm changes in acetylation on the histone level, and to associate histone acetylation patterns with the expression of genes of interest.

In queenright colonies, workers are mostly infertile due to queen-produced chemical signals such as pheromones [3–5]. Our data show that C646-treated workers remained in a “queenright-like state” and did not develop their ovaries following queen removal. Beyond their phenotypic similarities, C646-treated workers also shared the expression of many genes with workers from queenright colonies, as 33 and 40% of all differentially expressed genes in the queenright and the queenless group treated with C646 were commonly upregulated, respectively. We propose that this large overlap could be in part linked to the ant circadian rhythm since many of the commonly upregulated genes code for circadian clock-related proteins. On one hand, this is in line with a previous study in another *Temnothorax* species where the use of C646 led to the loss of the ability to adjust to new daily rhythms [36]. On the other hand, queen presence has been found to affect worker and colony activity in the honeybee [76, 77], which could explain the upregulation of circadian rhythm-related genes in workers from queenright colonies. Because both groups contain workers with less developed ovaries, we could alternatively hypothesize that worker sterility is maintained by the upregulation of genes with regulatory functions, which are then downregulated when workers become fecund following queen removal, explaining the large overlap of genes between the two groups.

The candidate gene “transketolase-like protein 2 isoform X2*”* was commonly downregulated in infertile workers and workers treated with the two chemical inhibitors, despite the relatively small number of genes (i.e., five) in the two lists of commonly downregulated genes. Transketolases are enzymes involved in the non-oxidative part of the pentose phosphate pathway (PPP) in all living organisms [53]. They are known to maintain low levels of reactive oxygen species (ROS) and are thus used in cancer treatment [54] and parasitic disease control [55]. More broadly, antioxidant production has been positively linked to lifespan in the fruit fly [78] and the nematode *Caenorhabditis elegans* [79], and has also been associated with long-lived ant queens [43].

## Conclusions

By experimentally manipulating histone (de)acetylation we show that this epigenetic mark might be required for workers to dynamically shift their physiology following queen removal. Our manipulation did not only affect life history traits such as fecundity, but also shifted the expression of genes with fecundity, immunity, and longevity functionalities. Our results thus provide insights into the molecular regulation of reproduction in social insects, which are prime examples of phenotypic plasticity.

## Methods

### Ant collection and maintenance

*Temnothorax rugatulus* ants are distributed throughout the western part of North America and reside in high elevation coniferous forests, under stones or in rock crevices. In August 2018, we collected colonies from nine different locations in the Chiricahua Mountains (Arizona, USA, Additional file 1 Table S1). In the laboratory, each colony was kept in a three-chambered box (9.7 x 9.7 x 2.9 cm) covered with a lid and containing an artificial nest made of a plastic insert between two glass slides covered by a red foil to block the light. The colonies were maintained at 21 °C and 70% humidity with a 12:12 light:dark cycle. They were fed weekly with half a cricket and a drop of honey and were provided with water ad libitum.

### Colony monitoring

We selected 90 monogynous colonies with 54 to 100 workers and reduced worker number to 50 per colony. To increase behavioral activity, colonies were then moved to a climate chamber at 25 °C and 70% humidity with a 12:12 light:dark cycle for two weeks. Before starting the experiment, colonies were randomly assigned to one of five experimental groups with a total of 18 colonies per group (Table 1).

**Table 1:** Group name, manipulation, treatment, and sample sizes (*N* = colonies at start / colonies for RNA-sequencing of fat body samples) for each experimental group. The queenless control was always used as a reference because it allows comparisons to all the other experimental groups differing in a single factor only i.e., queen presence or inhibitor treatment

Group name	Manipulation	Treatment	Colonies
Queenright	No queen removal	DMSO	*N* = 18 / 4
Queenless	Queen removal	DMSO	*N* = 18 / 5
Queenless+C646	Queen removal	DMSO+C646	*N* = 18 / 7
Queenless+TSA	Queen removal	DMSO+TSA	*N* = 18 / 4
Queenless+C646/TSA	Queen removal	DMSO+C646/TSA	*N* = 18 / 0

On the first day of the experiment, we removed all eggs, pupae, and males and adjusted the number of larvae to five per colony. Queens from the queen removal groups were removed and returned to their natal colonies. Then, colonies were fed with either the solvent DMSO (Carl Roth) only, the inhibitor of histone acetylation C646, which targets the p300/CPB histone acetyltransferases (50 μM in DMSO; Sigma-Aldrich) [80], the inhibitor of histone deacetylation TSA that inhibits class I and II histone deacetylases (50 μM in DMSO; Sigma-Aldrich) [81], or a combination of C646 and TSA (both 50 μM in DMSO). All preparations were diluted in 0.102 g/mL sucrose solution. The ants were fed for six weeks every other day with 15 μL of fresh solution per colony (Additional file 1 Fig. S9). Additionally, each colony received half a cricket every other day and water ad libitum. Once a week, we anesthetized all colonies with CO_2_. We removed and counted the eggs in queenless colonies to get precise numbers of worker-laid eggs. Once every two weeks, we counted all colony members (queens if applicable, workers, eggs, larvae, and pupae) in colonies from all groups.

We tested the effect of queen removal on worker survival by comparing worker number over time between the groups “queenright” and “queenless” using a linear mixed-effects model (LMM) with the package “lme4” [82]. In queenless colonies, we investigated the effect of treatment (DMSO, DMSO+C646, DMSO+TSA, and DMSO+C646/TSA) on worker number in interaction with time using a similar model. Colony identification (ID) was used as a random factor in both models to account for inter-colony variability. We assessed the fit of our LMMs using visual inspections of the residual distributions. The effect of treatment on egg production at week six was analyzed using a generalized linear mixed-effects model (GLMM, binomial family) with egg production as a binary variable. Our GLMM was tested for overdispersion using the package “DHARMa” [83]. All statistical analyses were conducted in R v3.5.1 [84].

### Experimental usage of chemical inhibitors

Depending on the doses, the chemical inhibitors C646 and TSA can have deleterious or beneficial effects on animals [85]. They have been repeatedly used in studies investigating plastic changes in ants, including *Temnothorax* ants, showing no negative effects in low concentrations [33, 36]. In a pilot experiment, we confirmed that the concentrations used in this study were indeed non-toxic for our ants. Accordingly, we did not observe an increased or decreased mortality following treatment with any of the chemical inhibitors (see results), or an upregulation of detoxification genes. Instead, we found a large overlap of upregulated genes between the “queenright” and “queenless+C646” groups (see results), indicating that the chemical inhibition of histone acetylation prevented workers to respond to queen removal, so that their gene expression resembles the one of workers from queenright colonies. In addition, our Principal Component Analysis (PCA) using all groups (see Additional file 1) does not indicate that samples from the chemical inhibitor treatments cluster together, as one might expect if toxins harmed the workers. Finally, other studies on *Temnothorax* ants using the same inhibitors reported increases in behaviors such as foraging or brood care following treatment [36; Marina Choppin and Philip Kohlmeier, pers. comm.] attesting to a general “well-being” of treated individuals. We therefore argue that the effects we report below are not mere side-products of the toxicity of the chemical inhibitors, but consequences of the inhibition of histone (de)acetylation.

### Dissections, RNA extractions, and fecundity measures

After six weeks, we selected a subset of colonies for dissections (Additional file 1 Methods). For each colony, we isolated all workers on or next to the brood pile in a Petri dish since brood-carers are typically close to the brood and usually the youngest individuals in the colony, thus more likely to develop ovaries following the loss of their queen [15, 86]. We dissected those workers on ice in a drop of a sterile saline solution until two workers with developed ovaries were found (2 to 14 workers dissected per colony). From these two workers, we cleaned the ovaries and took pictures for fecundity measurements using a stereomicroscope (Additional file 1 Dissection pictures) as detailed below. The fat bodies, including the first cuticle plate of the gaster, were collected from the two workers and pooled in the same Eppendorf tube containing 50 μl of TRIzol (Thermofisher) for further RNA-sequencing. Tissue collection took less than 10 min. The samples were flash-frozen in liquid nitrogen and preserved at −80 °C. Before the RNA extraction of each sample, we crushed the fat bodies with a pestle, added 50 μl of phenol:chloroform:isoamyl alcohol (25:24:1) (Carl Roth), mixed manually, and centrifuged at 1200 xg for 15 min. Afterward, the upper phase was transferred to a new tube and mixed with 25 μl of ethanol 100% (Carl Roth). RNA was then extracted using a NucleoSpin RNA XS kit (Macherey-Nagel). After quantity and quality control, 20 samples were sent for sequencing (Table 1). Library preparation was conducted following the standard protocol of BGI (Hongkong), which sequenced 150 bp paired-end reads on an Illumina Hiseq X Ten.

We measured ovariole length and counted the number of white eggs (i.e., eggs in development) and yellow bodies in the ovaries using the Leica software LAS v4.5. Yellow bodies are an indication of recent egg laying in ants [44–46]. We analyzed the effects of queen removal and treatment (DMSO, DMSO+C646, DMSO+TSA, DMSO+C646/TSA) on worker ovariole length using LMMs. We used GLMMs (binomial family) to test for effects of queen removal and treatment on the presence of white eggs and yellow bodies in the ovaries. Colony ID was used as a random factor to account for inter-colony variability. The models’ fit was assessed as described above.

### Gene expression analysis

Raw reads were trimmed with Trimmomatic v0.39 [87] (Additional file 1 Table S2) and quality checked using FastQC v0.11.7 [88]. The paired reads were then mapped against the *Temnothorax rugatulus* draft genome (Jongepier E, Alice S, Labutin A, Feldmeyer B, Gst C, Foitzik S: Convergent loss of chemoreceptors across independent origins of slave-making in ants, unpublished) using HISAT2 v2.1.0 [89] (Additional file 1 Table S2). We converted and sorted the output files using SAMtools v1.7 [90] and obtained a quality report from Qualimap v2.2.1 [91]. A genome-guided transcriptome assembly was created using StringTie v2.1.3 [92] and transcript sequences were extracted using GffRead v0.11.8 on the merged GTF file. Transcriptome quality was assessed using TransRate v1.0.3 [93]. Transcripts with an Open Reading Frame (ORF) < 100 bp were removed and the Python script “prepDE.py” from the online StringTie Manual was used to generate the gene count matrix (Additional file 4).

We assessed the effect of queen removal on worker gene expression by comparing the groups “queenless” and “queenright”. Then, we tested the effects of the chemical inhibition of histone acetylation and deacetylation by first comparing the groups “queenless” to “queenless+C646”, and then “queenless” to “queenless+TSA”. To avoid factitious DESeq2 results and for each comparison, we first filtered the gene count matrix so at least 70% of samples had a read count of ten or more reads per gene in at least one experimental group. We additionally plotted the maximum cook distance against the average gene expression per sample to identify and remove putative outliers. We used the filtered count matrix (Additional file 1 Table S3) to perform the differential gene expression analysis using DESeq2 [94]. An FDR-corrected *p*-value <0.05 was set as a significance threshold. We plotted principal component analyses (PCAs) with all genes using the package “ggplot2” [95] to assess the group-based clustering of our samples (Additional file 1 Fig. S10, S11, S12, S13). We created heatmaps with the package “pheatmap” [96] to visualize expression differences and clustering between samples. To annotate transcripts we conducted a BlastX homology search with BLAST v2.10.1+ [97] using the non-redundant invertebrate protein database from NCBI (May 2020) and only considered hits with an E-value <10^−5^ (Additional file 1 Methods). We combined the blast annotations with gene information from UniProt (www.uniprot.org). We chose to only discuss the top 15 most upregulated or downregulated genes based on adjusted *p*-values. Additionally, we used TransDecoder v5.5.0 [98] to translate nucleotide sequences into amino-acid sequences and then ran InterProScan v5.45–80.0 [99] to obtain Gene Ontology (GO) term annotations. Then, we performed a GO term enrichment analysis using the R package “topGO” [100] with the algorithm “weight01” (Additional file 1 Methods). We conducted the GO enrichment analysis separately for upregulated and downregulated genes in the groups compared to the queenless control. Statistical significance was given using Fischer exact tests. We extracted the overlap of upregulated and downregulated genes between the groups “queenright”, “queenless+C646” and “queenless+TSA” and assessed whether the overlap size between two groups was larger than expected by chance by resampling random gene lists (500 iterations). Finally, we plotted expression levels (i.e., normalized read counts) of genes of interest using “plotCounts” from DESeq2.

## Supplementary Information

**Additional file 1.** Contains additional tables (Table S1 to S3) and figures (Figure S1 to S13) related to the experimental design, the statistical analysis of worker number and fecundity, and the gene expression analysis, as well as additional methods and dissection pictures. Table and figure descriptions are included on the first page of the document.

**Additional file 2.** Contains the lists of upregulated and downregulated genes for each group comparison performed in our gene expression analysis, plus overlapping genes between groups.

**Additional file 3.** Contains the lists of GO terms associated with upregulated and downregulated genes for each group comparison performed in our gene expression analysis, with associated statistics from the enrichment analysis.

**Additional file 4.** Contains the original gene count matrix, obtained from the genome guided assembly, which was used in the gene expression analysis.

## Data Availability

Scripts and data sets for both the statistical analysis of worker number and fecundity and the gene expression analysis are available in a shared Google Drive folder at: https://drive.google.com/drive/folders/1f8pA772Yaogk8i-A1xiAiBRiFAsCohX8?usp=sharing. Raw reads for the transcriptomic analysis and associated metadata have been uploaded on the Short Read Archive (SRA) from NCBI under the BioProject PRJNA717637 (http://www.ncbi.nlm.nih.gov/bioproject/717637) and will be available after publication.

## Abbreviations

DEGs: Differentially expressed genes
DMSO: Dimethyl sulfoxide
FDR: False discovery rate
GO: Gene ontology
NCBI: National center for biotechnology information
TSA: Trichostatin A
