# Supplementary material for: Histone acetylation regulates the expression of genes involved in worker reproduction in the ant Temnothorax rugatulus
Source: BMC Genomics. 2021 Dec 3;22:871. doi: 10.1186/s12864-021-08196-8 (PMC8642982; doi:10.1186/s12864-021-08196-8)
Supplement: Supplementary file 1 — Additional file 1. Contains additional tables (Table S1 to S3) and figures (Figure S1 to S13) related to the experimental design, the statistical analysis of worker number and fecundity, and the gene expression analysis, as well as additional methods and dissection pictures. Table and figure descriptions are included on the first page of the document. [file 12864_2021_8196_MOESM1_ESM.docx]

**Supplemental Information for:**

**Histone acetylation regulates the expression of genes involved in worker reproduction in the ant *Temnothorax rugatulus***

Marina Choppin*, Barbara Feldmeyer and Susanne Foitzik

*correspondence: mchoppin

@uni-mainz.de

This document contains additional tables (Table S1 to S3) and figures (Figure S1 to S13) related to the experimental design, the statistical analysis of worker number and fecundity, and the gene expression analysis, as well as additional methods and dissection pictures.

| **Table S1** | Collection sites | Page 2 |
| --- | --- | --- |
| **Table S2** | Read trimming and mapping | Page 3 |
| **Table S3** | Filtered gene count matrices | Page 3 |

| **Figure S1** | Effect of queen removal on worker number | Page 4 |
| --- | --- | --- |
| **Figure S2** | Effect of queen removal on white eggs | Page 4 |
| **Figure S3** | Vitellogenin phylogeny | Page 5 |
| **Figure S4** | Effect of chemical inhibitors on worker number | Page 6 |
| **Figure S5** | Effect of chemical inhibitors on egg production | Page 6 |
| **Figure S6** | Effect of chemical inhibitors on white eggs | Page 7 |
| **Figure S7** | Heatmap DEGs TSA | Page 7 |
| **Figure S8** | Histograms resampling overlaps | Page 8 |
| **Figure S9** | Experimental timeline | Page 9 |
| **Figure S10** | PCA queen removal | Page 9 |
| **Figure S11** | PCA C646 | Page 10 |
| **Figure S12** | PCA TSA | Page 10 |
| **Figure S13** | PCA all groups | Page 11 |

**+ Methods** page 12

**+ Dissection pictures** page 13

**Table S1.** Collection sites, elevation, coordinates, and dates of collection.

| **Site** | **Elevation (m)** | **Coordinates** | **Dates of collection**  **(DD.MM.YYYY)** |
| --- | --- | --- | --- |
| H | 1976 | 31.909071; -109.253337 | 13.08.2018 & 20.08.2018 |
| I | 2607 | 31.919981; -109.277186 | 14.08.2018 |
| J | 1938 | 31.908966; -109.247523 | 14.08.2018 & 20.08.2018 |
| M | 2019 | 31.850508; -109.326524 | 16.08.2018 |
| N | 1951 | 31.855962; -109.329143 | 16.08.2018 |
| P | 1852 | 31.9127347; -109.2401991 | 15.08.2018 & 17.08.2018 |
| Q | 1787 | 31.916534; -109.232811 | 18.08.2018 |
| R | 1796 | 31.898606; -109.226421 | 20.08.2018 |
| S | 1935 | 31.897912; -109.241417 | 21.08.2018 – 26.08.2018  (except 23.08.2018) |

**Table S2.** Group information, number of raw reads, number and proportion of reads after trimming, and overall alignment rates after mapping for each sample.

| **Sample** | **Group** | **Raw reads** | **Reads post trimming** | **Overall alignment rate** |
| --- | --- | --- | --- | --- |
| 1 | queenright | 19912813 | 15515059 (77.91%) | 90.30% |
| 2 | queenright | 19116606 | 15874248 (83.04%) | 90.75% |
| 3 | queenright | 18320088 | 15555941 (84.91%) | 93.36% |
| 4 | queenright | 18319888 | 15501262 (84.61%) | 93.97% |
| 5 | queenless | 17523356 | 15481621 (88.35%) | 94.16% |
| 6 | queenless | 18320107 | 15976236 (87.21%) | 94.57% |
| 7 | queenless | 17504172 | 15347309 (87.68%) | 93.88% |
| 8 | queenless | 18320019 | 15960938 (87.12%) | 93.85% |
| 9 | queenless | 18319767 | 15599596 (85.15%) | 92.00% |
| 10 | queenless+C646 | 18320004 | 15657108 (85.46%) | 89.83% |
| 11 | queenless+C646 | 18320027 | 15902690 (86.80%) | 93.44% |
| 12 | queenless+C646 | 17523511 | 15409936 (87.94%) | 92.94% |
| 13 | queenless+C646 | 17523608 | 15776400 (90.03%) | 93.15% |
| 16 | queenless+C646 | 18192695 | 15473597 (85.05%) | 93.37% |
| 17 | queenless+C646 | 18320043 | 15682047 (85.60%) | 93.68% |
| 18 | queenless+C646 | 16502256 | 13977619 (84.70%) | 94.63% |
| 19 | queenless+TSA | 18320063 | 15743378 (85.94%) | 92.67% |
| 20 | queenless+TSA | 17523604 | 15485351 (88.37%) | 94.26% |
| 21 | queenless+TSA | 17523608 | 15555959 (88.77%) | 93.55% |
| 22 | queenless+TSA | 18320137 | 15547139 (84.86%) | 93.85% |

**Table S3.** Number of genes in the count matrix used for the differential expression analysis after filtering, for each comparison.

| **Group comparison** | **Filtered gene count matrix** |
| --- | --- |
| queenless vs queenright | 10791 genes |
| queenless vs queenless+C646 | 11036 genes |
| queenless vs queenless+TSA | 11022 genes |

**
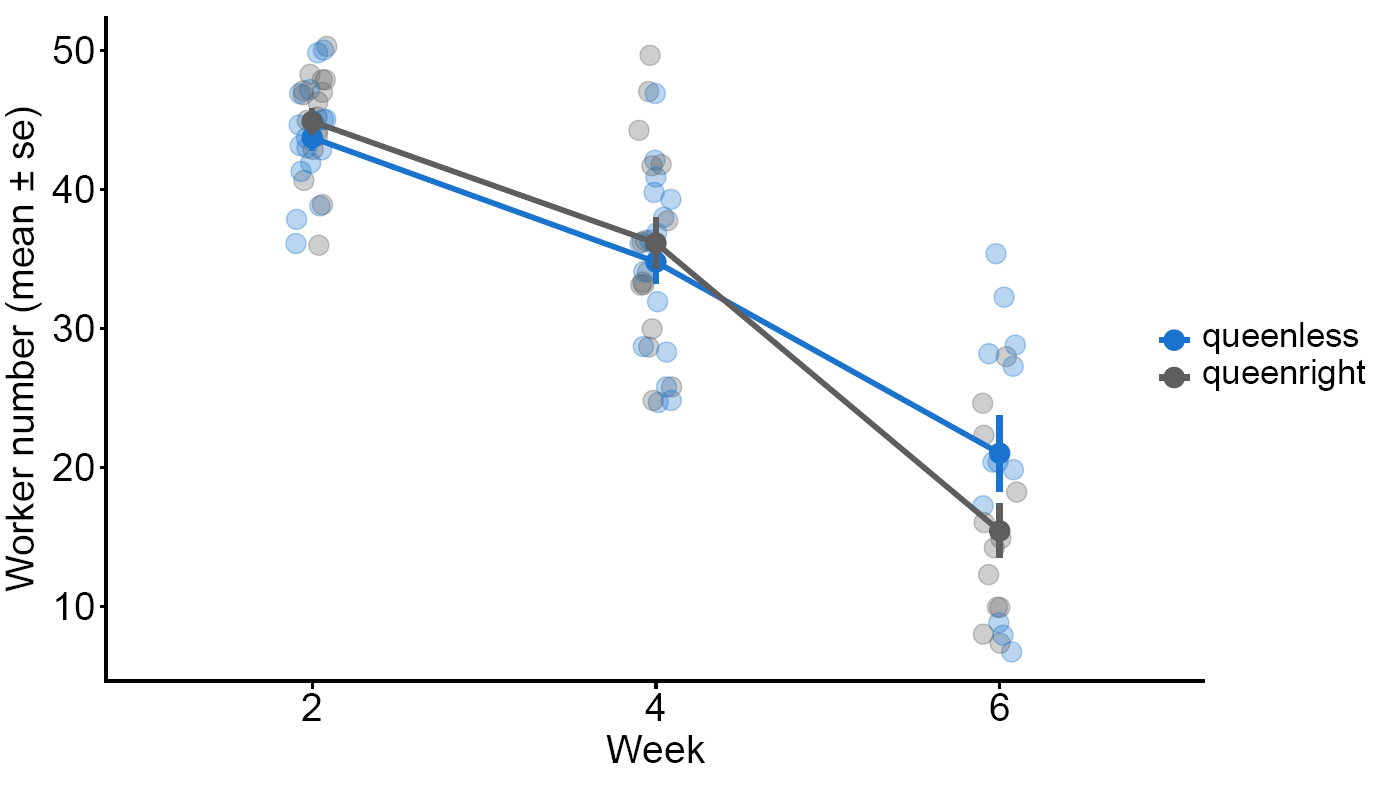
**

**Figure S1** Changes in worker number over the six weeks of experiment in the groups “queenless” (control, blue) and “queenright” (grey).


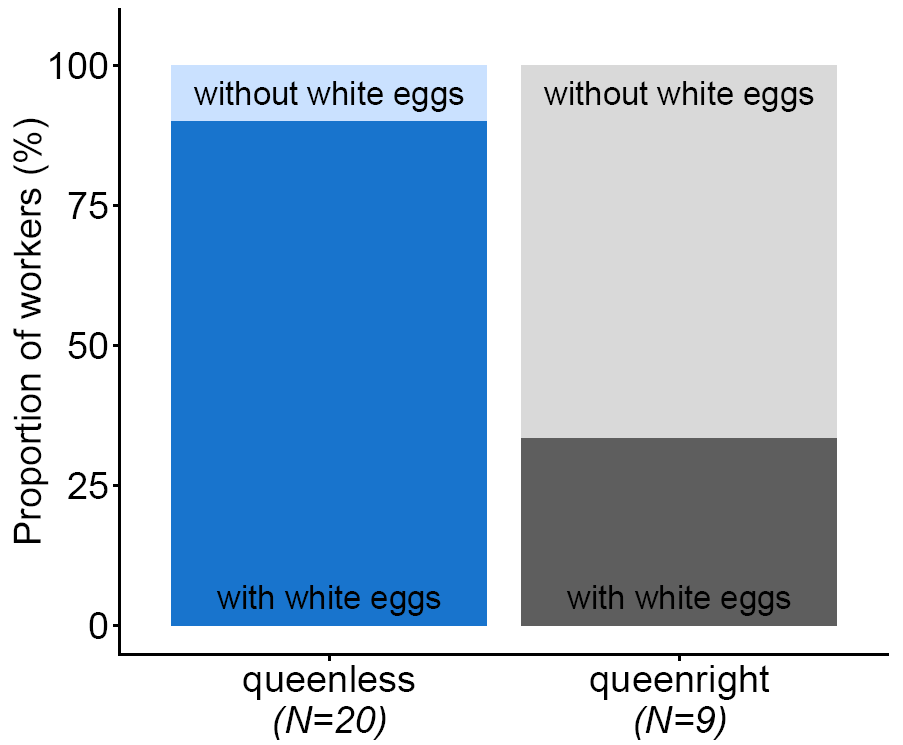


**Figure S2.** Proportion of workers with white eggs in the ovaries after six weeks of experiment in the groups “queenless” (control, blue) and “queenright” (grey).

**Figure S3.** Maximum Likelihood reconstruction of the vitellogenin phylogeny. The transcript ID of our vitellogenin copy is MSTRG.10425.2 and can be found in the blue cluster, marked in bold and with an asterisk.

**
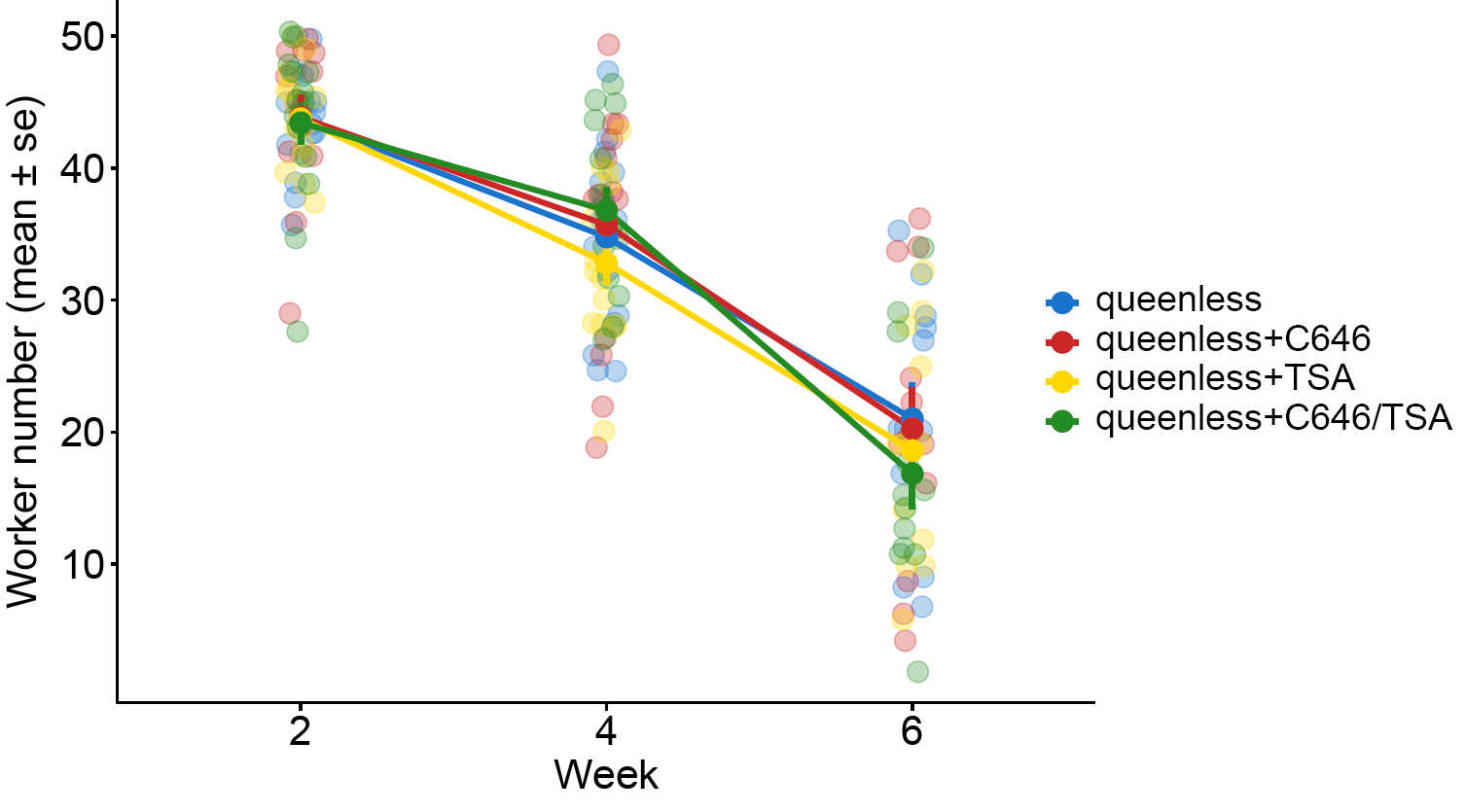
**

**Figure S4.** Changes in worker number over the six weeks of experiment in the groups “queenless” (control, blue), “queenless+C646” (red), “queenless+TSA” (yellow), and “queenless+C646/TSA” (green).


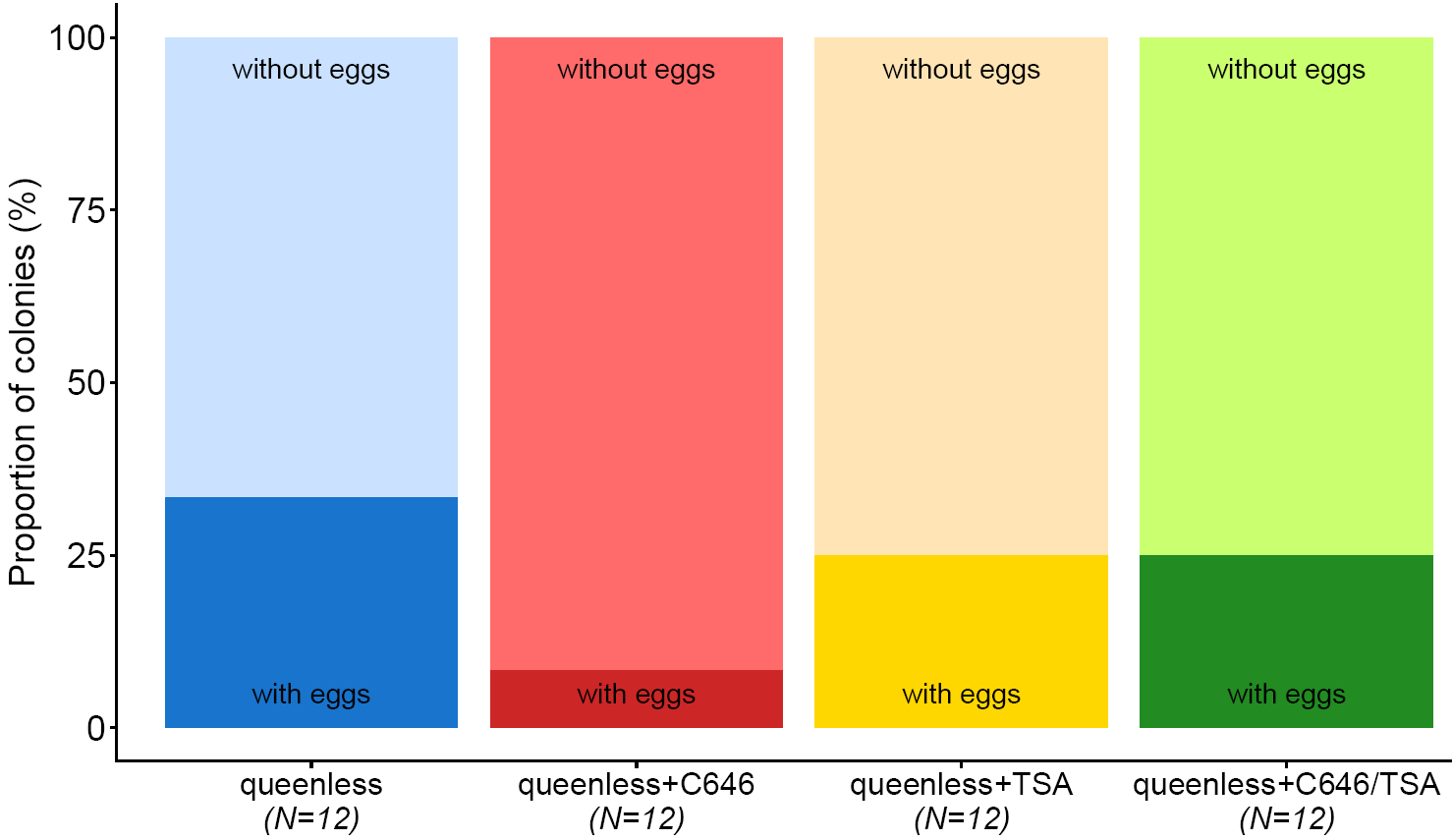


**Figure S5.** Proportion of colonies containing eggs after six weeks of experiment in the groups “queenless” (control, blue), “queenless+C646” (red), “queenless+TSA” (yellow), and “queenless+C646/TSA” (green).

**
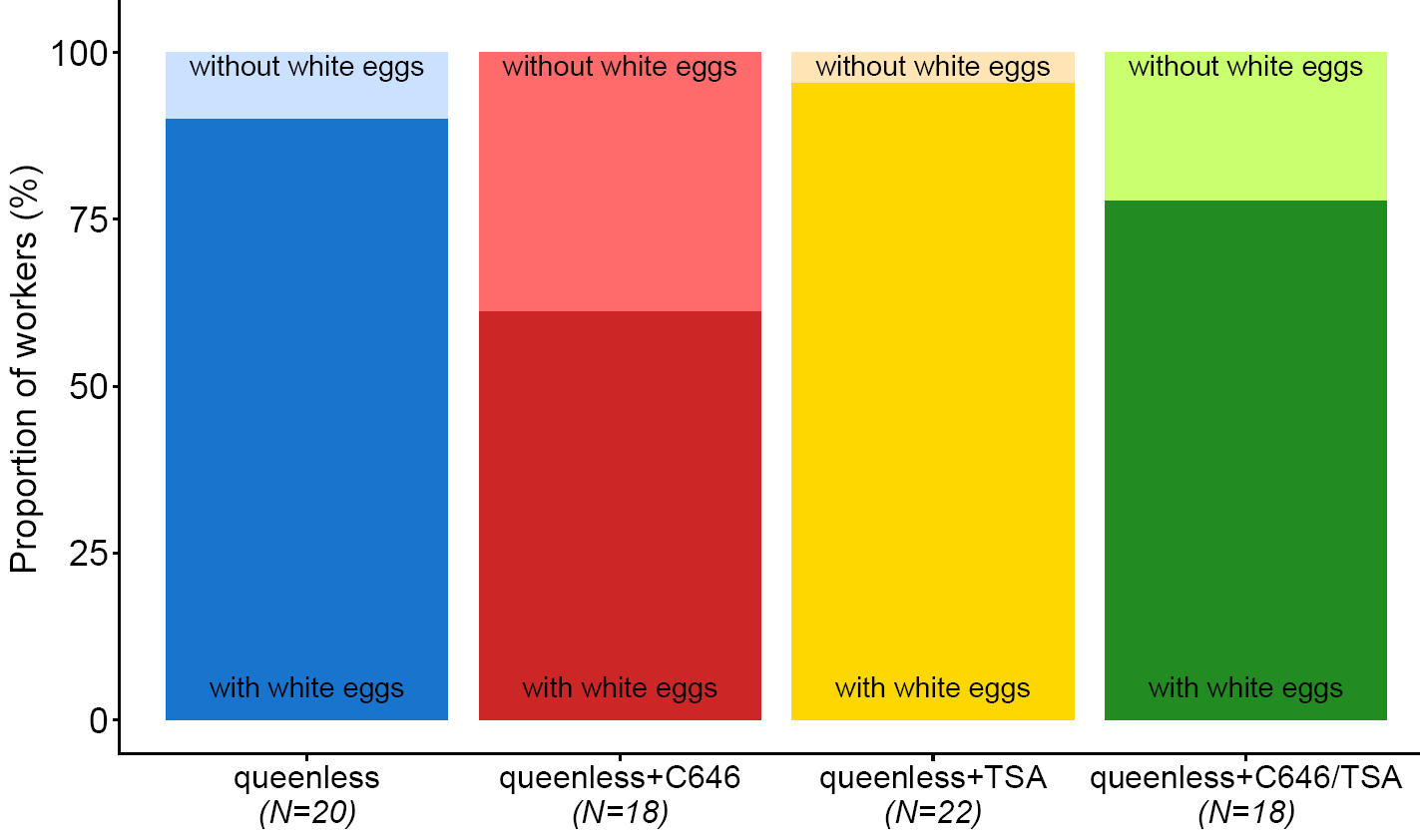
**

**Figure S6.** Proportion of workers with white eggs in the ovaries after six weeks of experiment in the groups “queenless” (control, blue), “queenless+C646” (red), “queenless+TSA” (yellow), and “queenless+C646/TSA” (green).


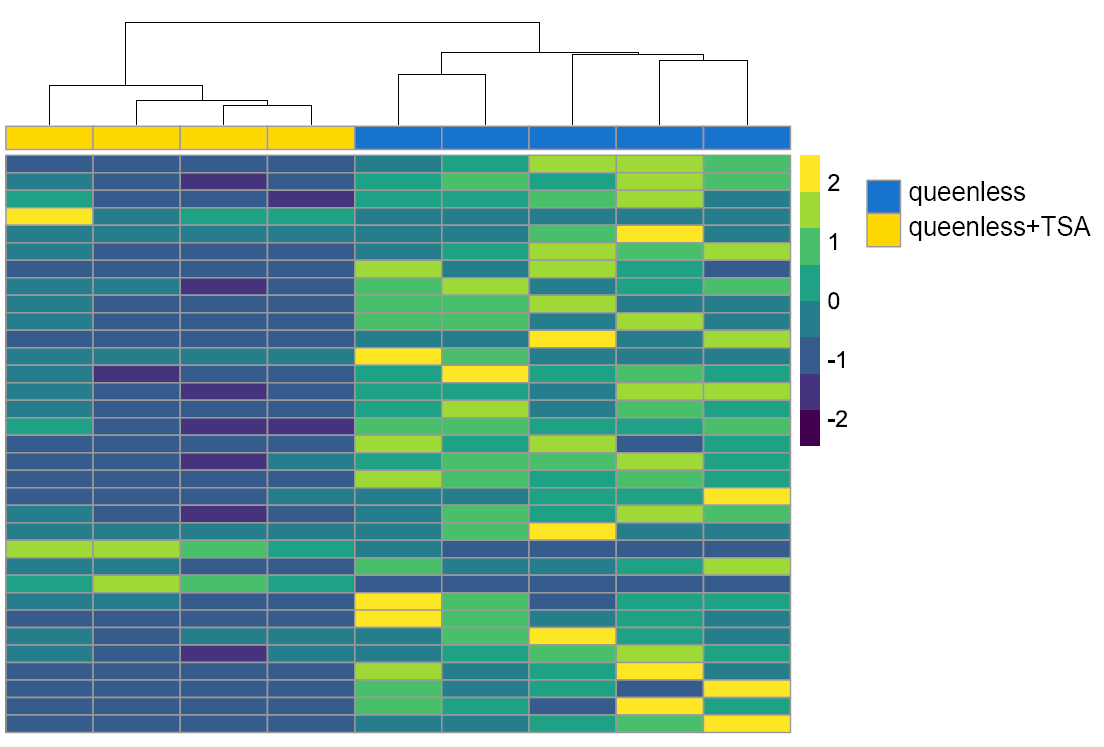


**Figure S7.** Heatmap showing the expression levels of differentially expressed genes between the groups “queenless” (control, blue) and “queenless+TSA” (yellow), and the clustering of samples per group.

**
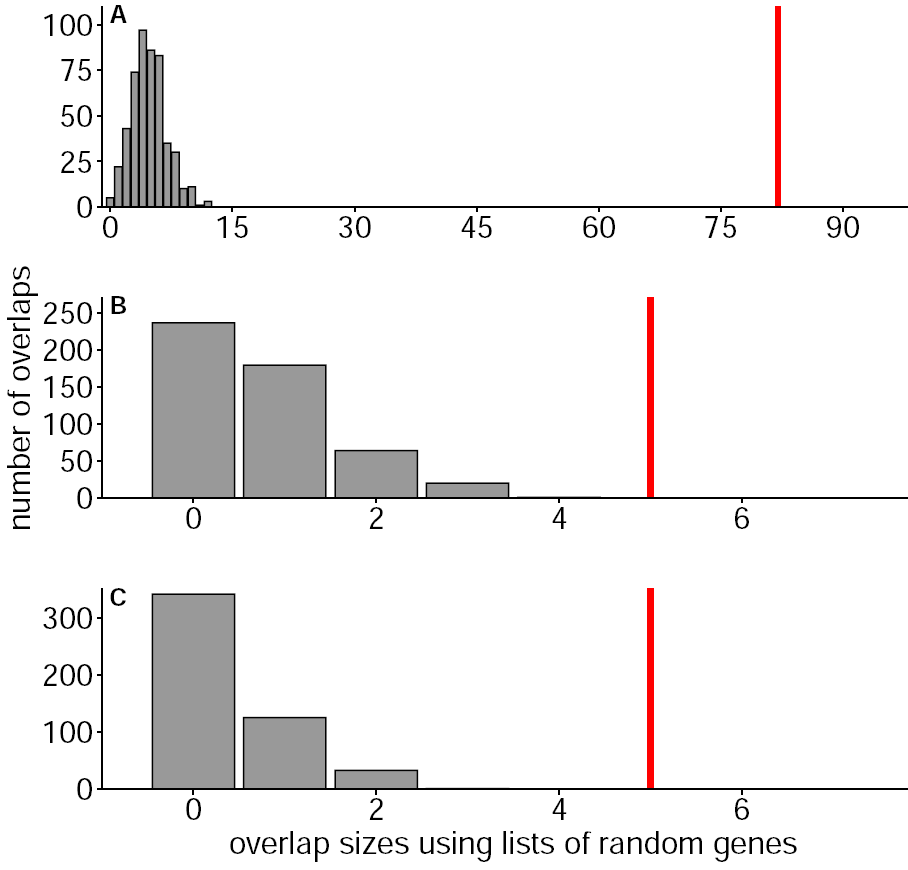
**

**Figure S8.** Distributions of overlap sizes obtained by resampling random gene lists (500 iterations, histograms) and actual number of overlapping genes (vertical red lines) between the groups “queenright” and “queenless+C646” (A, upregulated) (B, downregulated) and “queenright” and “queenless+TSA“ (C, downregulated)


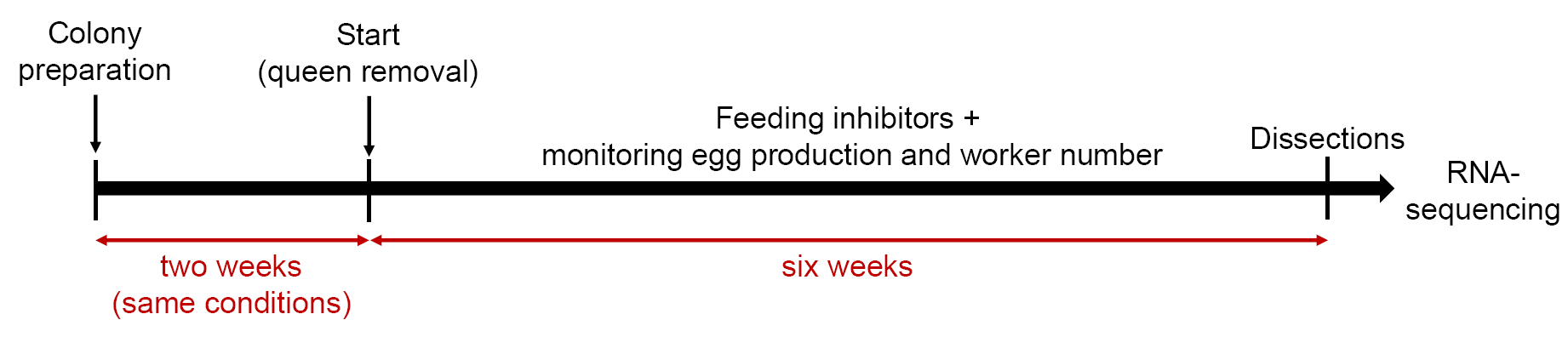


**Figure S9.** Experimental timeline.


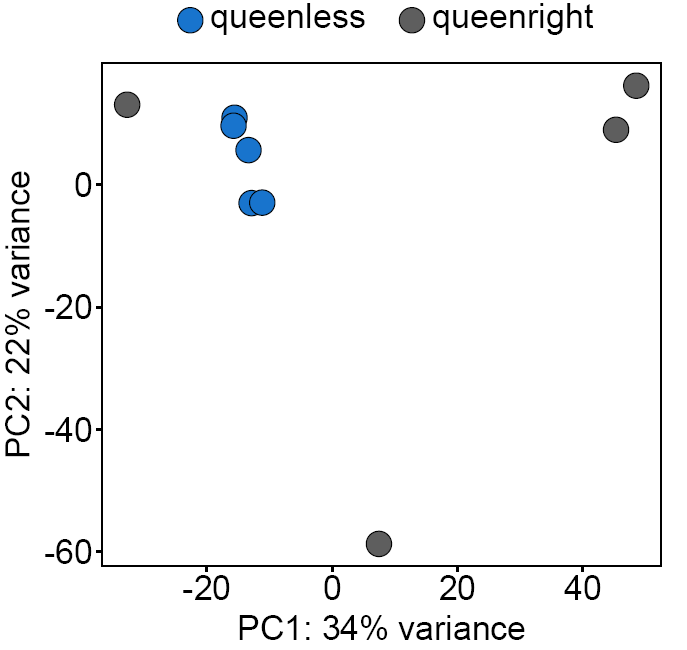


**Figure S10.** Principal Component Analysis (PCA) using all genes showing the clustering of samples from the groups “queenless” (control, blue) and “queenright” (grey).


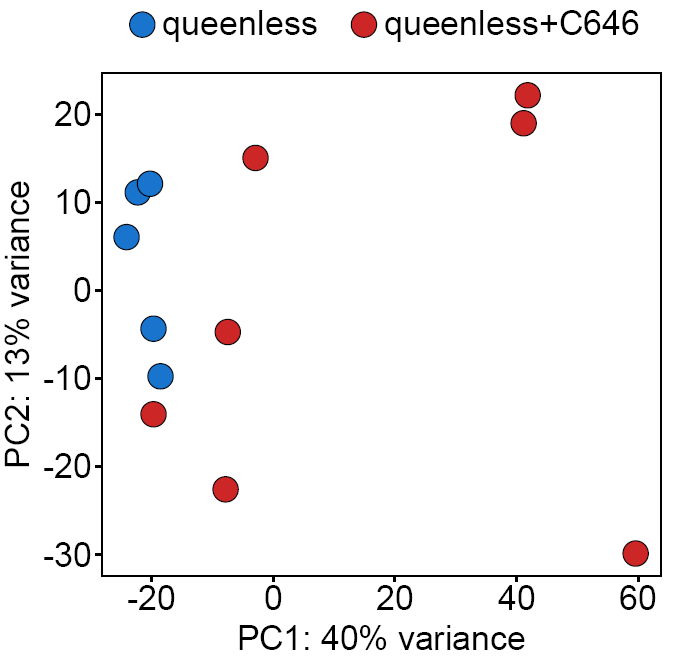


**Figure S11.** Principal Component Analysis (PCA) using all genes showing the clustering of samples from the groups “queenless” (control, blue) and “queenless+C646” (red).


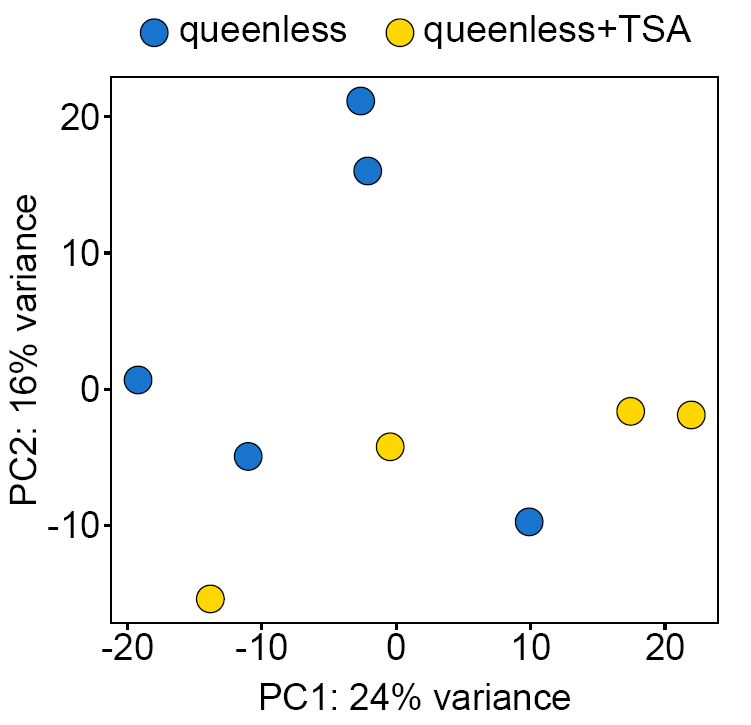


**Figure S12.** Principal Component Analysis (PCA) using all genes showing the clustering of samples from the groups “queenless” (control, blue) and “queenless+TSA” (yellow).


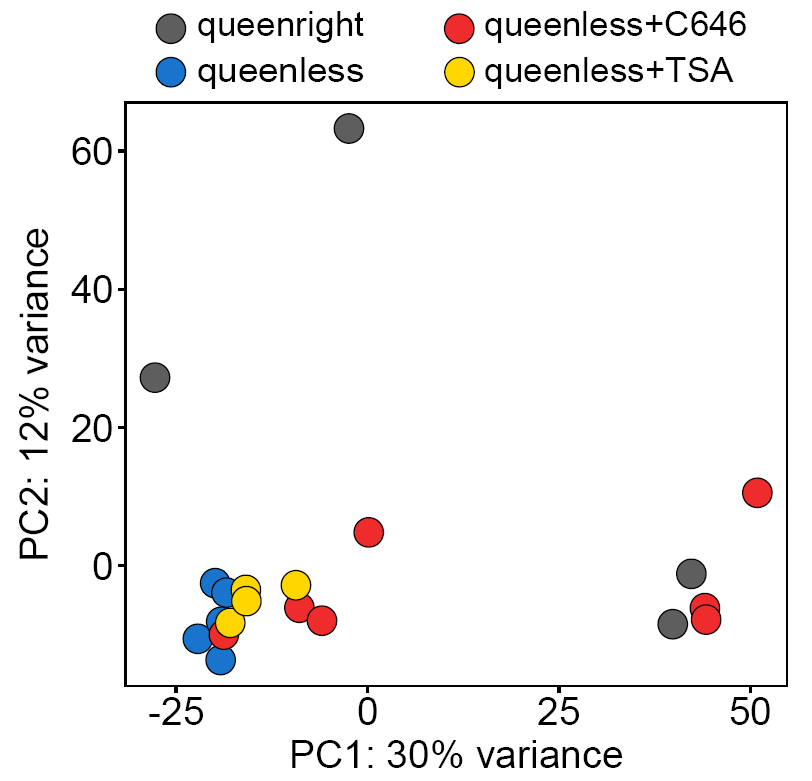


**Figure S13.** Principal Component Analysis (PCA) using all genes showing the clustering of samples from the groups “queenless” (control, blue), “queenright” (grey), “queenless+C646” (red), and “queenless+TSA” (yellow). All queenless samples from the control form a nice cluster which is most similar to the queenless+TSA samples. This is reflected in the rather small number of differentially expressed genes (i.e. 33) between these two groups. The queenless+C646 samples are somewhat heterogeneous with some workers reflecting the queenright, and others the queenless state. This might be the result of different amounts of inhibitor taken up by specific individuals and shows that the success and extent of the inhibition are variable but can be very strong using C646. We also see some variation in workers from the queenright colonies, probably due to different levels of ovary development among those workers, while most queenless workers develop their ovaries following the loss of their queen.

**Methods**

Selection of colonies for dissections

1) Assessing whether colonies are functional or not using the criteria below

Clear organization in the nest with presence of:

- at least ten workers

- the queen (for queenright colonies)

- larvae

- foragers outside

| **Group** | **Functional colonies** |
| --- | --- |
| queenright | N = 17 |
| queenless | N = 16 |
| queenless+C646 | N = 13 |
| queenless+TSA | N = 15 |
| queenless+C646/TSA | N = 14 |

2) For ovary dissections and fat body sampling we selected twelve colonies among the functional ones for which the total number of eggs produced over the six weeks of experiment was close to the average number of eggs produced by all colonies

Blast annotations

The BlastX homology search was done using the transcriptome obtained from the genome-guided assembly. To annotate our genes, we took for each transcript the hit with the smallest E-value and we assigned hits to genes that had more than one isoform transcript by selecting the longest isoform. We also added alternative blast hits for uncharacterized proteins, only if the percent identity was > 80%.

Enrichment analysis

For our enrichment analysis, we used Interproscan to obtain Gene Ontology (GO) term annotations associated with our transcripts. We then associated the GO term annotations with our genes. To do so, when a gene had more than one isoform transcript we combined the GO term annotations from its multiple isoform transcripts.

**Dissection pictures**

Pictures of worker ovaries at the end of the experimental period (six weeks post queen removal) for each experimental group. We selected pictures showing the mean ovariole length in workers of each experimental group.


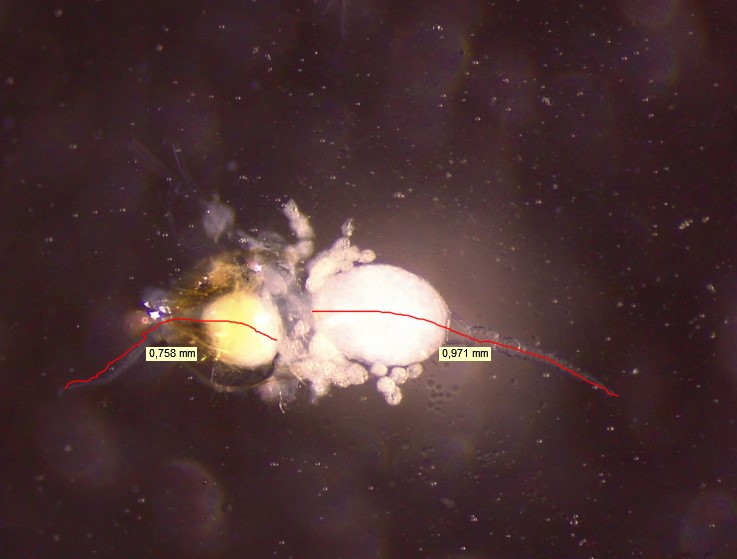

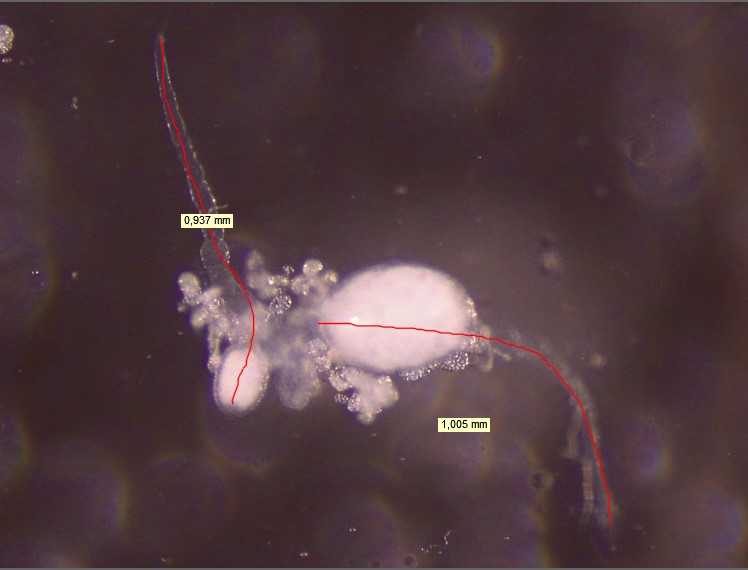

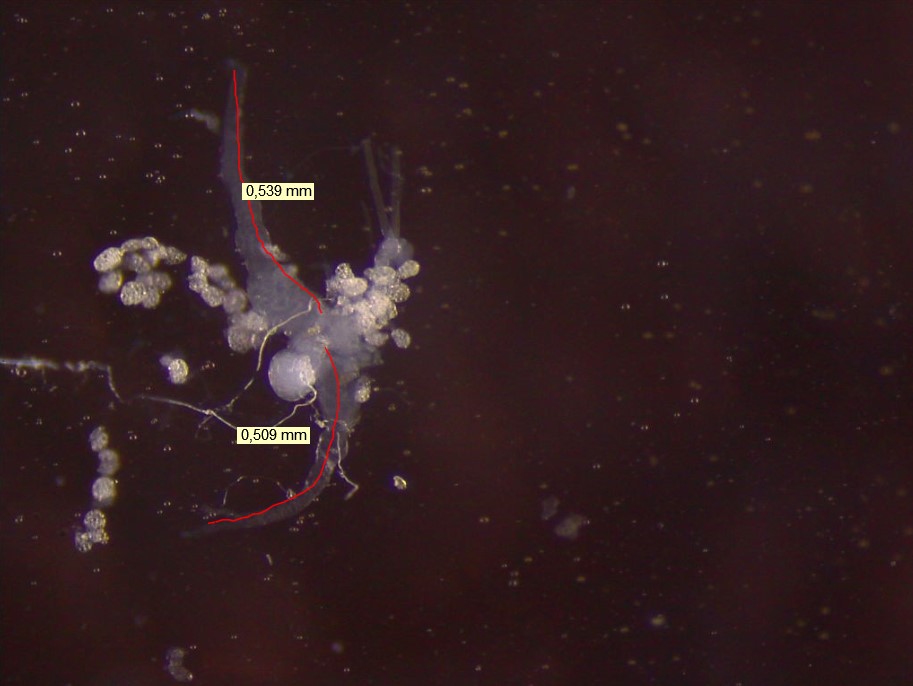
**
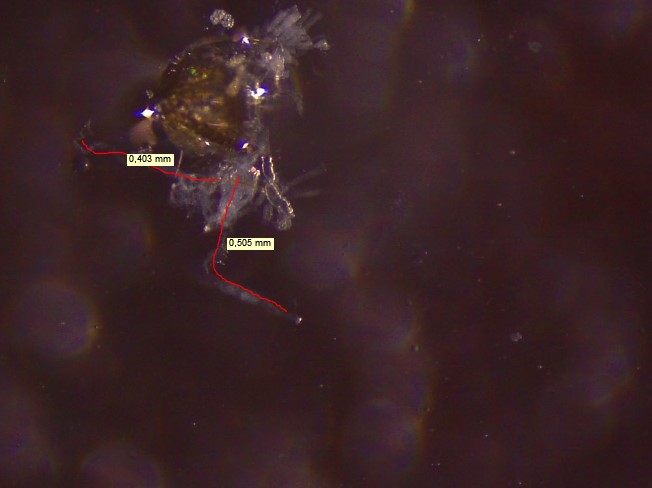
**
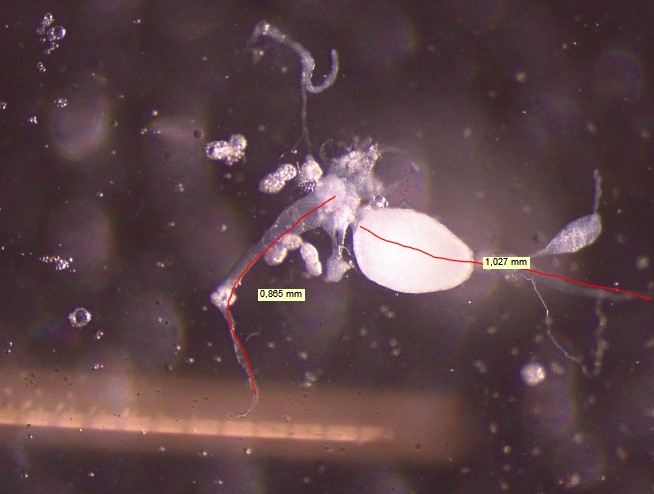


**queenright**

**queenless+TSA**

**queenless**

**queenless+C646/TSA**

**queenless+C646**
